# Determinants of adverse reactions to first-line antitubercular medicines: a prospective cohort study

**DOI:** 10.1186/s40545-023-00577-6

**Published:** 2023-06-08

**Authors:** Richard Delali Agbeko Djochie, Berko Panyin Anto, Mercy Naa Aduele Opare-Addo

**Affiliations:** Department of Pharmacy Practice, Faculty of Pharmacy and Pharmaceutical Sciences Kwame Nkrumah University of Science and Technology, Kumasi Private Mailbag, Kumasi, Ghana

**Keywords:** Antitubercular drugs, Adverse drug reactions, Pharmacovigilance, Ghana

## Abstract

**Background:**

The success of tuberculosis treatment relies on patients adhering to their medication regimen consistently. However, adherence levels tend to decrease among patients who experience adverse drug reactions to antitubercular medications, leading to suboptimal treatment outcomes. Hence, this study aimed to examine the types, incidence rates, and severity of adverse reactions caused by first-line antitubercular drugs. Additionally, it aimed to identify factors associated with the development of these reactions. By doing so, the study aimed to facilitate the provision of personalized and effective treatment to patients, ultimately improving treatment outcomes.

**Methods:**

Newly diagnosed patients with active tuberculosis were monitored from the start of their treatment until the completion of therapy. Any adverse reactions to anti-TB drugs that they encountered were carefully recorded. The collected data were analyzed using appropriate statistical methods such as analysis of variance, Chi-squared test, Fisher's exact test, and independent t-tests. Logistic regression was employed to assess the association between adverse drug reactions and various socio-demographic and clinical factors of the patients, using odds ratios as a measure of association.

**Results:**

Among the 378 patients included in the study, 181 individuals (47.9%) reported experiencing at least one adverse drug reaction, with an incidence rate of 1.75 events per 100-person months. The majority of these reactions occurred during the intensive phase of treatment. The gastrointestinal tract was the most commonly affected system, followed by the nervous system and skin. Patients aged over 45 years (OR = 1.55, 95% CI 1.01–2.39, p = 0.046) and those with extrapulmonary tuberculosis (OR = 2.41, 95% CI 1.03–5.64) were more likely to develop gastrointestinal reactions. Female gender was a significant predictor of both skin (OR = 1.78, 95% CI 1.05–3.02, p = 0.032) and nervous system (OR = 1.65, 95% CI 1.07–2.55, p = 0.024) reactions. Additionally, alcohol use and HIV infection were identified as independent predictors of adverse drug reactions affecting all three systems.

**Conclusion:**

Significant risk factors for developing antitubercular drug adverse reactions include alcohol consumption, cigarette smoking, being HIV positive, female gender and extrapulmonary tuberculosis.

**Supplementary Information:**

The online version contains supplementary material available at 10.1186/s40545-023-00577-6.

## Background

Tuberculosis (TB) remains a significant public health concern worldwide, particularly in developing countries with limited healthcare resources. It is the leading cause of death among individuals living with HIV/AIDS [1], with a rapid progression of the disease in this population [2]. Antibiotic chemotherapy is the fundamental approach to combat TB, and successful treatment outcomes rely on patients maintaining high levels of adherence to their anti-TB medications. In Ghana, the treatment of TB aligns with the World Health Organization's (WHO) recommendations, utilizing a combination of rifampicin, isoniazid, pyrazinamide, and ethambutol. The dosages are calculated based on the patient's weight during the initial 2 months (intensive phase), followed by a continuation phase of rifampicin and isoniazid for an additional 4 months. Most patients undergoing this treatment regimen achieve sputum sterility within 2 weeks, thereby reducing the risk of transmitting the infection in their communities.

The tuberculosis treatment success rate in Ghana has shown improvement over the years, increasing from 50% in 2000 to 84% in 2012 [3]. Aligned with the WHO's End TB Strategy, the Ministry of Health in Ghana aimed to further enhance the treatment success rate to 91% for all forms of TB by 2020. However, as of the end of 2022, the treatment success rate remained at 84% [4]. Nonadherence to treatment is a significant contributing factor to unsuccessful TB treatment outcomes, with approximately half of TB patients failing to complete their treatment. This leads to prolonged infectiousness, relapse, and an increased risk of mortality [5]. In Ghana, studies have indicated treatment adherence rates ranging from 62 to 63% [6, 7], and these studies have revealed that patients who experience adverse drug reactions or have concerns about medication side effects are less likely to adhere to their treatment plans [7–9]. These adverse drug reactions play a major role in nonadherence and can result in significant complications, the development of multi-drug resistant TB, relapse, and even death [10, 11].

Adverse reactions to antitubercular drugs are a prevalent issue during tuberculosis (TB) treatment, contributing to treatment failure, increased morbidity, and mortality. Nevertheless, there is a lack of research investigating the factors that contribute to these adverse reactions among TB patients in Ghana. This study aims to contribute to the existing knowledge by exploring the sociodemographic and clinical determinants of adverse reactions to antitubercular drugs among TB patients in Ghana, a lower-middle-income country. The study findings will assist policymakers in developing a management algorithm for tuberculosis adverse drug reactions (ADRs), enabling healthcare professionals to readily identify TB patients who are at a higher risk of experiencing ADRs. This identification would facilitate proper monitoring and provide more personalized and effective treatment, ultimately improving treatment outcomes and achieving high rates of treatment adherence.

## Aim

The objective of this study was to examine the various types of adverse reactions caused by first-line antitubercular drugs, determine their incidence rates, assess their severity, and identify the factors associated with the development of these reactions.

## Methods

### Study design and participants

This prospective observational study was carried out in eight tuberculosis treatment centres in the Eastern and Ashanti Regions of Ghana. The study sites consisted of three primary care hospitals and one referral hospital in each region. These sites were chosen because they consistently reported the highest number of TB cases annually in their respective regions in the past 5 years.

The study population included all newly diagnosed drug-susceptible TB patients who were ready to start TB treatment. However, only those who were 18 years or older, have access to a mobile phone and agreed to participate by signing a consent form were included while psychiatric patients, those diagnosed with liver or kidney impairment, and those pregnant were excluded.

### Data collection and analysis

The data collection tool was designed using REDCap, guided by the study objectives. The tool was validated and pretested in a hospital that was not involved in the main study. The responses from the pretest helped to modify the tool accordingly and to determine the prevalence of ADR among the study population for sample size estimation for the main study.

Participants were contacted via telephone calls at least once a week to inquire about the incidence of any untoward effect from their TB medication from initiation until they completed their treatment, lost to follow-up or died between January 2021 and June 2022. Any adverse reaction that they reported at the clinic and documented in their TB treatment card was also included in the data analysis. The data collection process took place during the peak of the COVID-19 outbreak in Ghana, necessitating the implementation of several safety measures to ensure the well-being of both researchers and participants. As part of these measures, participants and data collectors were provided with face masks, which they wore consistently throughout the study. Additionally, during each visit to the clinic for medication refills, social distancing guidelines were followed, and hand sanitiser was made readily available.

An antitubercular ADR was defined as any negative or unexpected side effect that occurs when a patient is taking standard, first-line drugs i.e. isoniazid, rifampicin, pyrazinamide and ethambutol used to treat tuberculosis. Participants were screened using a list of side effects associated with antitubercular drugs that have been documented in the literature. The Naranjo ADR Probability Scale was used to deduce a causal relationship between the ADR reported and the antitubercular drugs. A causal relationship was confirmed in the following instances: when a patient continued to experience the same reaction after a re-challenge with the drug, when the reaction only occurred following administration of the drug, when the reaction subsided or ceased upon administration of an antidote, and when the patient was not using any alternative medication known to cause the same reaction [12]. Only ADRs classified as *Certain* (a score of ≥ 9), *Probable* (a score of 5–8) or *Possible* (a score of 1–4) were used in the analysis.

Participants' antitubercular ADRs were assessed for severity using Hartwig's ADR severity assessment scale. The ADRs were categorized as severe (levels 5, 6, and 7) if they directly resulted in death, in-patient or prolonged hospitalization. ADRs were classified as moderate (levels 3 and 4) if they necessitated the withholding or discontinuation of anti-TB treatment and/or required an antidote. Mild ADRs (levels 1 and 2) were characterized by the need to withhold anti-TB drugs without the use of an antidote [13, 14]. To facilitate analysis, moderate-to-severe ADRs were grouped as *major ADRs*, while mild ADRs were considered *minor ADRs.*

Data cleaning was performed using Microsoft Excel 2016, and subsequent analysis was conducted using Statistical Package for the Social Sciences (SPSS) version 21. Appropriate statistical tests, such as analysis of variance, Chi-squared, Fisher's exact, and independent t-tests, were utilized. The results were presented as frequencies, means, and standard deviations. Logistic regression was employed to identify socio-demographic and clinical factors associated with the development of antitubercular ADRs, reporting both crude odds ratios and adjusted odds ratios. Multivariate analysis was conducted when significant crude odds ratios were observed. Statistical significance was determined at a 5% level, and results were reported with a 95% confidence interval.

## Results

### Sociodemographic characteristics

The study involved 378 participants who were diagnosed with drug-susceptible tuberculosis with a mean (SD) age of 45.3 (15.1) years (Range: 18–91 years). The majority of participants were males (67.2%), employed (76.5%), had basic education (60.9%), and were diagnosed with pulmonary TB (92.6%). Only 6.4% of participants smoked tobacco, while 25.4% said they drink alcohol and 25.7% had HIV co-infection (Table 1). More than half of the participants were unmarried (single, divorced, or widowed). The mean (SD) baseline weight of study participants was 52.6 (10.7) kg, and the median (IQR) was 50.0 (45–58) kg.

**Table 1:** Socio-demographic and clinical characteristics of study participants

Characteristics	Number of patients *n*	Percentage of total
Age (years)		
18–25	26	6.9
26–35	84	22.2
36–45	90	23.8
46–55	90	23.8
56–65	49	13.0
≥ 66	39	10.3
Gender		
Male	254	67.2
Female	124	32.8
Marital status		
Married	167	44.2
Single	127	33.6
Divorced	41	10.9
Widowed	43	11.3
Employment status		
Employed	289	76.5
Unemployed	89	23.5
Educational level		
Basic	230	60.9
Secondary	70	18.5
Tertiary	29	7.7
None	49	12.9
Type of TB		
Pulmonary TB	350	92.6
Extra-pulmonary TB	23	6.1
Both	5	1.3
Smoking status		
Smoker	24	6.4
Nonsmoker	354	93.6
Alcohol us**e**		
Yes	96	25.4
No	282	74.6
HIV status		
HIV positive	97	25.7
HIV negative	275	72.8
HIV status unknown	6	1.5

### Incidence, duration and severity of antitubercular adverse drug reactions

**Fig. 1:**
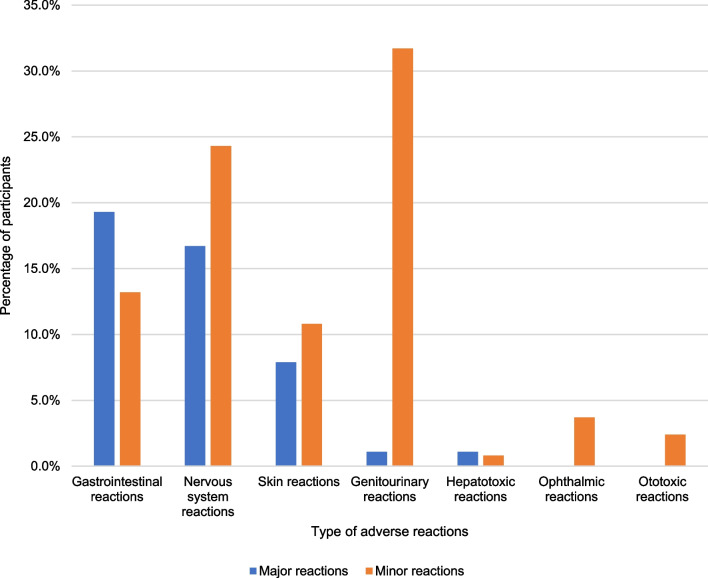
Type and severity of antitubercular drug adverse reactions reported by study participants

Out of the 378 patients who were observed for 51,730 person-months, 181 individuals (47.9%) experienced at least one adverse drug reaction with a total of 904 events. This translated into an ADR incidence rate of 1.75 events per 100-person months. Most of the major ADRs occurred in the gastrointestinal tract, with 73 events reported by 19.3% of patients, followed by the nervous system with 63 events reported by 16.7% of patients, and the skin with 30 events reported by 7.9% of patients, as shown in Fig. 1. The average time for gastrointestinal (GI) symptoms to manifest was around 12 days, with a duration of 13 days. The duration of abdominal pain was found to be longer in smokers compared to nonsmokers (56.0 days vs 9.3 days; p < 0.001). HIV-positive individuals also experienced longer durations of abdominal pain compared to HIV-negative individuals (15.5 days vs 6.8 days; p = 0.023), particularly among those who delayed antiretroviral treatment (19.6 days vs 6.6 days; p = 0.014). Furthermore, smoking (30.2 days vs 11.4 days; p = 0.006), having pulmonary TB (13.8 days vs 4.6 days; p < 0.001), and being male (16.1 days vs 8.0 days; p = 0.003) were associated with a delay in the occurrence of vomiting. On the other hand, nausea occurred earlier in patients with extrapulmonary TB compared to those with pulmonary TB (2.4 days vs 9.0 days; p = 0.004).

As shown in Table 2, the incidence of nervous system adverse reactions varied between 0.04 events per 100 person-months (seizures) and 6.43 events per 100 person-months (numbness), typically occurring approximately 1 month after initiating treatment. Participants with pulmonary TB and smokers experienced a significantly prolonged delay before experiencing numbness, while numbness lasted longer in females and HIV-positive individuals. The onset of myalgia was delayed in males, and its duration was extended in smokers and participants with extrapulmonary TB. Peripheral neuritis appeared earlier in HIV-positive individuals compared to those without HIV (27.3 days vs 63.7 days; p = 0.011), and the duration of arthralgia was longer in females compared to males (40.0 days vs 18.4 days; p = 0.023), as well as in participants aged 45 years or older.

**Table 2:** Incidence of adverse drug reactions by persons and person months

Adverse effects		Person months	Number of Persons (%)	Number of events	Incidence^†^	95% CI
Gastrointestinal reactions	Nausea	1925	60 (15.9)	60	3.12	2.42–4.01
	Vomiting	1945	58 (15.3)	58	2.98	2.30–3.86
	Anorexia	1900	65 (17.2)	65	3.42	2.68–4.36
	Abdominal pain	1989	50 (13.2)	50	2.51	1.91–3.32
	Diarrhoea	2011	46 (12.2)	46	2.29	1.71–3.05
Nervous system reactions	Ataxia	2238	6 (1.6)	6	0.27	0.12–0.60
	Numbness	1665	107 (28.3)	107	6.43	5.32–7.77
	Dizziness	1807	82 (21.7)	82	4.54	3.76–5.76
	Impaired concentration	2258	2 (0.5)	2	0.09	0.02–0.35
	Slurred speech	2247	4 (1.1)	4	0.18	0.07–0.50
	Seizure	2264	1 (0.3)	1	0.04	0.01–0.31
	Peripheral neuritis	2226	10 (2.6)	10	0.45	0.24–0.84
	Myalgia	1832	76 (20.1)	80	4.37	3.51–5.44
	Arthralgia	1794	86 (22.8)	90	5.02	4.08–6.17
Skin reactions	Rash	2172	17 (4.5)	17	0.78	0.49–1.26
	Pruritus	1914	62 (16.4)	64	3.34	2.62–4.27
	Photosensitive skin	2258	2 (0.5)	2	0.09	0.02–0.35
Genitourinary reactions	Urine discolouration	1593	115 (30.4)	124	7.78	6.53–9.28
	Oliguria	2256	2 (0.5)	2	0.09	0.02–0.35
Hepatotoxic reactions	Hepatitis	2260	2 (0.5)	2	0.09	0.02–0.35
	Jaundice	2229	7 (1.9)	7	0.31	0.15–0.66
Ophthalmic reactions	Blurred vision	2204	13 (3.4)	13	0.59	0.34–1.02
	Reduced visual acuity	2254	3 (0.8)	3	0.13	0.04–0.41
Ototoxic reactions	Tinnitus	2240	5 (1.3)	5	0.22	0.09–0.54
	Hearing problem	2249	4 (1.1)	4	0.18	0.07–0.47

^†^Incidence is expressed as number of events per 100-person months. CI: confidence interval

Pruritus, which was the most commonly experienced skin reaction affected 16.4% of participants, with an incidence rate of 3.32 events per 100-person months (Table 2). Skin rash affected 4.5% of patients, with an incidence rate of 0.78 events per 100-person months. The latent period for pruritus was 8.6 days (95% CI 4.6–12.7) from the start of treatment, and it lasted almost 2 weeks. The duration of pruritus was prolonged in HIV-positive participants (p = 0.025). Other patient and clinical factors did not appear to be statistically associated with skin reactions.

Hepatotoxic responses were only recorded in 2.4% of study subjects, and the only genitourinary ADR reported was urine discolouration which affected about one-third of participants. With just 16 and 9 incidents, respectively, the incidence of ocular and ototoxic ADRs was equally low and of moderate severity. None of the ADRs reported by participants resulted in the termination of TB treatment, but there were brief withdrawals and administration of antidotes.

### Factors associated with developing a major antitubercular ADR

As depicted in Table 3, participants who were over 45 years old had an odds ratio (OR) of 1.55 with a 95% confidence interval (CI) ranging from 1.01 to 2.39, and a p-value of 0.046, indicating that they were more likely to experience a gastrointestinal adverse effect. Similarly, participants who consumed alcohol had an OR of 2.76 with a 95% CI ranging from 1.71 to 4.56, and a p-value of less than 0.001, indicating a higher likelihood of developing a GI adverse effect. Furthermore, HIV-positive participants had the highest OR of 3.12 with a 95% CI ranging from 1.93 to 5.06, and a p-value of less than 0.001, indicating that they were most likely to experience a GI adverse effect.

**Table 3:** Odds of developing adverse drug reactions affecting various systems in association with clinical characteristics

Characteristics	GI reactions		Nervous system reactions		Skin reactions		Hepatotoxic reactions	
	OR	95% CI	OR	95% CI	OR	95% CI	OR	95% CI
Age > 45 years (versus ≤ 45 years)	**1.55**	**1.01**–**2.39**	1.36	0.90–2.05	1.37	0.82–2.31	1.13	0.28–4.57
Female gender (versus male)	1.36	0.86–2.13	**1.65**	**1.07**–**2.55**	**1.78**	**1.05**–**3.02**	1.55	0.34–7.03
Cigarette Smokers (versus nonsmokers)	0.84	0.34–2.09	1.76	0.77–4.05	1.48	0.57–3.88	–*	–
HIV-positive (versus HIV-negative or NA)	**3.13**	**1.93**–**5.06**	**3.81**	**2.34**–**6.20**	**2.76**	**1.60 -4.75**	3.90	0.86–17.75
Alcohol use (versus no alcohol)	**2.76**	**1.71**–**4.46**	**2.74**	**1.71**–**4.41**	**2.67**	**1.55**–**4.60**	0.41	0.05–3.41
Extra-pulmonary TB (versus pulmonary TB)	**2.41**	**1.03**–**5.64**	1.95	0.12–0.83	–	–	–	–

OR: odds ratio; CI: confidence interval; HIV: human immunodeficiency virus; NA: not available; GI: gastrointestinal; TB: tuberculosis. Boldface entries indicate statistically significant associations. ^*^ insufficient numbers so estimates are unreliable

Logistic regression (odds ratio) was used to compare the demographic variables with the likelihood of developing an adverse drug reaction. Aged 45 years and above, HIV positive, alcohol use and extrapulmonary TB had increasing odds of developing GI reaction with odds ratios 1.55, 3.13, 2.76, and 2.41 respectively. Females, HIV positive and alcohol use also had increasing odds of developing nervous system reactions with odds ratios of 1.65, 3.81, and 2.71 respectively. Sociodemographics had no statistical relation with hepatotoxic reactions. Skin reactions had statistical relations with female gender (OR: 1.78), HIV positive (OR: 2.76) and alcohol use (OR: 2.67). In summary, alcohol use and HIV positive have a strong statistical relationship with the development of adverse reactions except for hepatotoxic reactions

We also found that certain factors were independent predictors of nervous system adverse drug reactions. Cigarette smoking had an odds ratio (OR) of 1.76 with a 95% confidence interval (CI) ranging from 0.77 to 4.05, and a p-value of 0.1796, indicating that it was not significantly associated with a nervous system adverse reaction. On the other hand, alcohol use had an OR of 2.74 with a 95% CI ranging from 1.71 to 4.41, and a p-value of less than 0.001, indicating a significant association with a nervous system adverse reaction. Finally, being HIV-positive had the highest OR of 3.81 with a 95% CI ranging from 2.34 to 6.20, and a p-value of less than 0.001, indicating a significant association with a nervous system adverse reaction.

Furthermore, the results indicate that the female gender, alcohol use, and being HIV-positive were predictors of developing a skin reaction. The female gender had an OR of 1.78 with a 95% CI ranging from 1.05 to 3.02, and a p-value of 0.032, indicating a significant association with the development of a skin reaction. Similarly, alcohol use had an OR of 2.67 with a 95% CI ranging from 1.55 to 4.60, and a p-value of 0.0005, indicating a significant association with the development of a skin reaction. Finally, being HIV-positive had an OR of 2.76 with a 95% CI ranging from 1.60 to 4.75, and a p-value of 0.0003, indicating a significant association with the development of a skin reaction.

## Discussion

The incidence rate of adverse drug reactions found in the current study was 1.75 events per 100-person months, and nearly half of the participants (47.9%) experienced at least one ADR. This incidence rate was higher compared to a Canadian study [15], which only focused on major ADRs and reported an incidence rate of 1.48. The proportion of patients developing ADRs was similar to findings from Iran [16] and India [17]. It is worth noting that the variability in the incidence of ADRs related to tuberculosis drugs is influenced not only by factors like race or geography but also by the type of study conducted. Retrospective studies that relied on voluntary patient reports tended to record lower rates compared to prospective studies [18].

The majority of ADRs (96.9%, n = 876) experienced by study participants occurred during the intensive treatment phase, which aligns with findings from a study conducted in Brazil [19]. This result was expected, considering that the intensive phase of TB treatment typically involves a higher number of drugs compared to the continuation phase. The administration of multiple drugs can lead to drug–drug interactions, particularly with anti-TB regimens containing rifampicin, which affects the metabolism of concurrent medications. In the continuous phase, the side effects of ADRs appeared to diminish, possibly due to the auto-induction of enzymes, resulting in lower plasma concentrations of rifampicin [20].

The most common ADRs observed in the current study primarily affected the gastrointestinal system, the nervous system, and the skin. Gastrointestinal ADRs encompassed symptoms such as nausea, vomiting, abdominal pain, and diarrhoea. The incidence of gastrointestinal ADRs identified in this study was comparable to findings from Korea [18] but higher than those reported in Malaysia [21]. In terms of ADRs affecting the hepatobiliary system, the proportion of patients experiencing such reactions in this study was lower compared to a Brazilian study [19]. This discrepancy may be attributed to the Brazilian study's inclusion of ADRs detected through laboratory tests in their data analysis, whereas the current study focused solely on clinically observed ADRs. The skin-related adverse drug reactions documented in this study predominantly included pruritus (itching) and rash. These types of skin reactions are commonly associated with medications such as pyrazinamide, isoniazid, rifampicin, and the antiretroviral regimen used for HIV treatment in coinfected patients [22, 23].

More than 40% of patients (n = 155) experienced at least one adverse drug reaction (ADR) affecting the nervous system, although the severity of most of them was found to be mild to moderate. The incidence rate of nervous system ADRs documented in this study was higher compared to rates of 7.1% in Korea [18] and 1.1% in Malaysia [21]. Among the participants, over one-fifth (n = 82; 21.7%) reported experiencing dizziness, with an incidence rate of 4.54 events per 100-person months. This incidence rate exceeded that reported in the Korean and Malaysian studies [18, 21]. Dizziness, which can be attributed to medications such as isoniazid and ethambutol [24, 25], poses a risk of falls, particularly among older individuals, increasing the likelihood of bone fractures and associated morbidity and mortality. Adequate monitoring of elderly TB patients is crucial to prevent or manage such ADRs. Additionally, arthralgia and myalgia were observed as nervous system ADRs in this study, affecting 22.8% and 20.1% of patients, respectively, at rates of 5.02 and 4.37 events per 100-person months. The severity of these symptoms only necessitated analgesic treatment without interrupting TB treatment.

Isoniazid and ethambutol are the primary medications used in TB treatment that can cause ADRs affecting the nervous system [26]. Ethambutol is known to potentially induce reversible optic neuritis. On the other hand, isoniazid can lead to peripheral neuropathy by inhibiting pyridoxine-phosphokinase, an enzyme responsible for converting pyridoxine to pyridoxal 5-phosphate. This conversion is essential for pyridoxine-mediated metabolisms, including those of proteins, carbohydrates, and various compounds such as brain amines [27]. As a result, peripheral neuropathies may occur. However, this can be prevented by supplementing with a daily dose of 50 mg of pyridoxine [26, 27]. In Ghana, the standard practice is to provide pyridoxine supplementation to patients with an increased risk of neuropathy. However, it was found that healthcare staff did not routinely assess the risk of neuropathy for most patients. Pyridoxine supplements were only given to patients when they started experiencing symptoms of neuropathy. This practice could explain the high incidence of numbness observed in this study. It is crucial to enhance provider education on the appropriate use of pyridoxine supplementation to ensure that patients receive its benefits promptly.

Alcohol use and HIV infection were identified as risk factors for developing ADRs affecting the gastrointestinal system, skin, and nervous system. Additionally, the incidence of gastrointestinal ADRs was associated with older age (> 45 years), while nervous system and skin reactions were associated with females and tobacco use, respectively. It is important to note that alcohol consumption increases the risk of ADRs, particularly in women [28]. Alcohol can interfere with the absorption and metabolism of medications when taken concurrently, leading to an increased incidence of ADRs [29, 30].

Isoniazid, a crucial component of the TB treatment regimen in both the intensive and continuation phases, undergoes rapid metabolism in the presence of alcohol. This interaction can result in treatment failure, isoniazid-associated hepatotoxicity, and disulfiram-like reactions [31]. The higher incidence of ADRs observed in alcohol consumers could be attributed to this interaction between alcohol and antitubercular drugs. Therefore, it is crucial to provide counselling to newly diagnosed TB patients, advising them to avoid alcohol or reduce its consumption to acceptable levels of less than 14 units per week [32] during treatment. This approach helps mitigate the risk of developing ADRs and allows patients to benefit from the cardiovascular health advantages associated with reduced alcohol consumption.

The cumulative toxicities and potential interactions between HIV drug regimens and anti-TB drugs put patients at risk of experiencing recurrent adverse drug reactions (ADRs) [23, 33]. The role of HIV in the development of ADRs in TB, as observed in this study, aligns with findings from Canada [15], Nigeria [34], the UK [35], and Brazil [19].

The association between older age and ADR development has been extensively explored and established in the literature [36–38]. As individuals age, physiological changes occur in the body, even in the absence of chronic diseases. These changes can impact the pharmacokinetic profiles of medications, including factors such as volume of distribution, metabolism, and rate of excretion, ultimately leading to prolonged half-life and increased toxicities [39].

Females, in general, tend to be more susceptible to ADRs compared to their male counterparts [40–44]. In Ghana, the anti-TB drug regimen consists of a single pill containing four medications (during the intensive phase) or two medications (during the continuation phase) to enhance adherence, making individualized dosing challenging. Consequently, healthcare professionals rely on weight bands to determine the appropriate doses, which may introduce inaccuracies. However, males typically have more muscle mass and heavier bones than females, resulting in healthy men often weighing more than healthy women of the same height. Since the administration of TB drugs in the study sites is typically based on weight bands, this could potentially lead to females receiving higher doses of anti-TB drugs, thereby increasing the incidence of ADRs, as observed [45]. This discrepancy may explain the higher incidence of ADRs reported in females compared to males.

Active tobacco smokers have a higher risk of experiencing extrapulmonary TB, as well as severe recurrent pulmonary TB [46], and increased susceptibility to developing adverse drug reactions (ADRs) from anti-TB medications [17, 47, 48]. Therefore, it is essential to provide counselling to TB patients who smoke, encouraging them to quit smoking. By doing so, they can not only avoid the occurrence of such ADRs but also benefit from improved cardiovascular health and reduced risks of cancer associated with smoking cessation [49, 50].

It was observed that patients with extrapulmonary TB had more than twice the likelihood of developing gastrointestinal ADRs (p < 0.05) and nearly twice the likelihood of developing nervous system ADRs (p > 0.05) compared to those with pulmonary TB. Although a causal relationship was not established, similar findings have been reported in previous studies [34, 51]. Consequently, it is crucial to monitor extrapulmonary TB patients adequately, facilitating timely diagnosis and appropriate management of these ADRs.

## Further research

Considering that TB treatment outcomes go beyond microbiologic cure, it is essential to examine the impact of these ADRs on patients' health-related quality of life and overall treatment outcomes. This broader perspective will contribute to expanding the scientific community's knowledge in this field.

## Strengths and weaknesses

This study represents one of the recent endeavours in Ghana aimed at evaluating adverse reactions associated with antitubercular drugs and exploring their contributing factors. The findings of this study will provide valuable insights for healthcare practitioners to identify vulnerable patients and promptly diagnose and treat their adverse drug reactions. By incorporating a relatively higher number of study sites (8), participants from diverse backgrounds were monitored, enhancing the generalizability of the findings. However, it is important to note that the study sites only included primary and secondary hospitals, thereby excluding TB patients receiving treatment from lower-level health facilities like health centres or higher-level facilities such as tertiary hospitals. Furthermore, due to financial constraints, laboratory tests to assess the impact of anti-TB drugs on renal, cardiovascular, and hepatic enzymes could not be conducted. Therefore, future studies that encompass patients from a broader range of health facilities and incorporate laboratory assessments of adverse drug reactions will provide additional perspectives to the discussion surrounding antitubercular adverse drug reactions.

## Conclusion

Approximately half of the participants experienced at least one adverse drug reaction, with the gastrointestinal tract being the most commonly affected, followed by the nervous system and skin. A majority of these ADRs occurred during the intensive phase of treatment. Significant risk factors for developing adverse reactions to antitubercular drugs include alcohol consumption, cigarette smoking, being HIV positive, being female, and having extrapulmonary tuberculosis. Therefore, healthcare providers treating tuberculosis must identify these vulnerable patient groups to prevent, diagnose, and manage these ADRs. By doing so, patients can adhere to treatment and achieve higher cure rates.

## Supplementary Information

**Additional file 1.** Manuscript dataset.

## Data Availability

The datasets generated and analysed during the current study are included in this published article as a Additional file 1.

## Abbreviations

ADR: Adverse drug reaction
HIV: Human immunodeficiency virus
TB: Tuberculosis
WHO: World Health Organization
